# Supplementary material for: Lung Cancer and Self-Management Interventions: A Systematic Review of Randomised Controlled Trials
Source: Int J Environ Res Public Health. 2022 Jan 4;19(1):536. doi: 10.3390/ijerph19010536 (PMC8744740; doi:10.3390/ijerph19010536)
Supplement: Supplementary file 1 [file ijerph-19-00536-s001.zip › ijerph-1455827-supplementary.pdf]

**Table S1: Socio-demographic Variables.**	
**Socio-Demographic Variable**	**Background**
Age	The median age for a lung cancer diagnosis is 70 years. Most trials are made up of younger subjects, which makes the extrapolation of younger patient data to older patients less meaningful [15]. Further, multimorbidity increases with age [16] which could influence participation and outcomes of SM interventions.
Sex	Men are more likely to develop lung cancer compared to women, with global ratios of 1 in 18 men and 1 in 51 women developing the disease between birth and 79 years [17].
Education	Not completing high school is associated with poorer health outcomes for several diseases [18]
Smoking status	Smoking is the biggest risk factor for lung cancer, accounting for approximately 85% of lung cancer cases. Risk increases with the number of cigarettes smoked and the number of years smoked. Risk declines in subjects who stop smoking and the risk improves the longer subjects remain smoke free. Lung cancer is associated with never smokers in around 15-20% of cases often associated with genomic aberrations and female sex [15]. Continued cigarette smoking after a lung cancer diagnosis is associated with a poorer QoL [19]
relationship/living arrangement	Evidence suggests that being married has a desirable effect on cancer mortality and patterns of care [20]. Patients with cancer that live alone are found to have higher incidence and mortality rates [21]

1. Chen, H.m., et al., *Randomised controlled trial on the effectiveness of home-based walking exercise on anxiety, depression and cancer-related symptoms in patients with lung cancer.* The British Journal of Cancer, 2015. **112**(3): p. 438-445.
2. Chen, H.M., et al., *Effect of walking on circadian rhythms and sleep quality of patients with lung cancer: a randomised controlled trial.* Br J Cancer, 2016. **115**(11): p. 1304-1312.
3. Edbrooke, L., et al., *Multidisciplinary home-based rehabilitation in inoperable lung cancer: a randomised controlled trial.* Thorax, 2019. **74**(8): p. 787.
4. Edbrooke, L., et al., *Benefits of home-based multidisciplinary exercise and supportive care in inoperable non-small cell lung cancer - protocol for a phase II randomised controlled trial.* BMC Cancer, 2017. **17**(1).
5. Liu, Z., et al., *Two-week multimodal prehabilitation program improves perioperative functional capability in patients undergoing thoracoscopic lobectomy for lung cancer: A randomized controlled trial.* Anesthesia and Analgesia, 2020. **131**(3): p. 840-849.
6. Mills, M.E., et al., *Does a Patient-Held Quality-of-Life Diary Benefit Patients With Inoperable Lung Cancer?* Journal of Clinical Oncology, 2009. **27**(1): p. 70-77.
7. Schofield, P., et al., *A tailored, supportive care intervention using systematic assessment designed for people with inoperable lung cancer: A randomised controlled trial.* Psycho-Oncology, 2013. **22**(11): p. 2445-2453.

8. Wangnum, K., et al., *Impact of the multidisciplinary education program in self-care on fatigue in lung cancer patients receiving chemotherapy.* Journal of the Medical Association of Thailand = Chotmaihet thangphaet, 2013. **96**(12): p. 1601-1608.
9. Yount, S.E., et al., *A randomized trial of weekly symptom telemonitoring in advanced lung cancer.* Journal of Pain and Symptom Management, 2014. **47**(6): p. 973-989.
10. Zhang, L.-L., et al., *Tai Chi Exercise for Cancer-Related Fatigue in Patients With Lung Cancer Undergoing Chemotherapy: A Randomized Controlled Trial.* Journal of Pain and Symptom Management, 2016. **51**(3): p. 504-511.
11. Zhu, X., et al., *Influence of self-management exercise intervention on the cancer related fatigue severity and self-management efficacy of patients with non-small cell lung cancer after operation.* JPMA. The Journal of the Pakistan Medical Association, 2020. **70 [Special Issue]**(9): p. 88-93.
12. Chen, H.M., et al., *Effect of walking on circadian rhythms and sleep quality of patients with lung cancer: A randomised controlled trial.* British Journal of Cancer, 2016. **115**(11): p. 1304-1312.
13. Yount, S.E., et al., *A randomized trial of weekly symptom telemonitoring in advanced lung cancer.* J Pain Symptom Manage, 2014. **47**(6): p. 973-89.
14. Zhu, X., et al., *Influence of self-management exercise intervention on the cancer related fatigue severity and self-management efficacy of patients with non-small cell lung cancer after operation.* JPMA. The Journal of the Pakistan Medical Association, 2020. **70**(9): p. 88-93.
15. Ganti, A.K. and D.E. Gerber, *Lung Cancer*. 2013, Cary, UNITED STATES: Oxford University Press, Incorporated.
16. Divo, M.J., C.H. Martinez, and D.M. Mannino, *Ageing and the epidemiology of multimorbidity.* The European respiratory journal, 2014. **44**(4): p. 1055-1068.
17. McIntyre, A. and A.K. Ganti, *Lung cancer—A global perspective.* 2017. **115**(5): p. 550-554.
18. Pincus, T., et al., *Social conditions and self-management are more powerful determinants of Health than access to care.* Annals of internal medicine, 1998. **129**(5): p. 406-411.
19. Clark, M.M., et al., *Motivational readiness for physical activity and quality of life in long-term lung cancer survivors.* Lung Cancer, 2008. **61**(1): p. 117-122.
20. Gomez, S.L., et al., *Effects of marital status and economic resources on survival after cancer: A population-based study.* Cancer, 2016. **122**(10): p. 1618-1625.
21. Elovainio, M., et al., *Living alone as a risk factor for cancer incidence, case-fatality and all-cause mortality: A nationwide registry study.* SSM - Population Health, 2021. **15**: p. 100826.
